# Editorial: Interactions between autophagy and immune response: cell communication and disease implications

**DOI:** 10.3389/fimmu.2025.1676537

**Published:** 2025-08-21

**Authors:** Gustavo J. S. Pereira, Veronica Bordoni, Manuela Antonioli

**Affiliations:** ^1^ Department of Pharmacology, Escola Paulista de Medicina, Universidade Federal de São Paulo, São Paulo, Brazil; ^2^ Department of Cell and Developmental Biology, University College London, London, United Kingdom; ^3^ Department of Hematology/Oncology, Cell and Gene Therapy, Bambino Gesù Children’s Hospital (IRCCS), Rome, Italy; ^4^ Department of Epidemiology, Preclinical Research and Advanced Diagnostics, National Institute for Infectious Diseases “Lazzaro Spallanzani”- IRCCS 00149, Rome, Italy; ^5^ Department of Biology, University of Rome “Tor Vergata”, Rome, Italy

**Keywords:** autophagy, immune system, cellular mechanism, cell communication, pathology, therapy

Autophagy, a key cellular pathway that promotes the recycling to maintain the homeostasis, has also emerged as a central player in shaping immune responses across diverse physiological and pathological conditions. Indeed, this mechanism is increasingly recognized as more than a degradative pathway for cellular homeostasis; it is now understood to be a central regulator of intercellular communication, particularly in modulating immune responses. Through its involvement in the secretion of cytokines (*e.g.*, IL-1β, IL-6, CXCL2) and in the biogenesis of extracellular vesicles, autophagy intersects with both innate and adaptive immunity. These secretory functions, often overlapping with apoptotic and non-canonical pathways, suggest that autophagy governs not only intracellular protein quality control but also long-range immune modulation. However, the complexity of autophagy-driven paracrine and distal signalling still remains poorly explored, especially in disease contexts such as cancer, infection, and neurodegeneration. In the Research Topic we have recently presented, a compelling narrative has emerged: from transplantation immunology to autoimmunity, cancer, neuroinflammation, and haematological malignancies, autophagy potentially interfaces with immune cells to modulate disease progression and therapeutic outcomes.

In this context, the original research article by Wang et al. investigated the molecular basis of acute rejection following liver transplantation, highlighting how JNK signalling enhances CD8^+^ T cell autophagy, thereby exacerbating immune-mediated graft injury. Inhibition of JNK disrupts BECN1 activation and limits the autophagic response, ultimately reducing CD8^+^ T cell proliferation and improving transplant outcomes. This work not only advances our understanding of rejection biology but also identifies potential therapeutic targets that modulate T cell function via the autophagy pathway. Further exploring the immunomodulatory roles of autophagy in T cells, Zhao et al. reviewed several recent evidence about its significance in autoimmune diseases, where this pathway has been demonstrated to regulate T cell activation, differentiation, and survival. In this regard, aberrant autophagic activity is linked to dysregulated immune responses in systemic lupus erythematosus and rheumatoid arthritis, thereby highlighting the dual role of autophagy, which is both protective and pathogenic, depending on the cellular context and disease stage. Hence, autophagy becomes a double-edged sword that must be finely tuned to maintain immune homeostasis. Whereas Zhao et al. focus on T cells, Wang et al. examine the impact of autophagy on B cell biology, offering a complementary perspective. Their review delineates the roles of both canonical and noncanonical autophagy in B cell development, antigen presentation, and memory formation. Notably, B cell function relies on autophagy not only for survival and plasma cell differentiation but also for effective germinal centre reactions. Moreover, autophagy dysregulation in B cells contributes to autoimmune pathologies, suggesting that autophagy modulation may be therapeutically relevant across multiple immune cell types. In contrast, Lee et al. delve into the underexplored territory of macrophage extracellular traps (METs), uncovering the molecular machinery that enables viable macrophages to release DNA-based traps without undergoing cell death. The process relies on Gasdermin D pore formation at the nuclear envelope, triggered by the extracellular cold-inducible RNA-binding protein via TLR4 signalling. Once in the cytoplasm, nuclear DNA is trafficked through the autophagic-lysosomal pathway and secreted as METs complexes enriched with histones and myeloperoxidase. This discovery adds a new layer of complexity to macrophage biology and suggests that autophagy-dependent mechanisms play a role in inflammatory diseases and cancer immunity.

This cellular-level perspective is expanded by the work of Rodolfo and Campello, who explore how extracellular vesicles (EVs), including exosomes and the recently described migrasomes, participate in intercellular communication within the tumour microenvironment (TME). These migration-released vesicles, often released by tumour or stromal cells, can transport autophagy-related signals that influence immune evasion and therapeutic resistance. Migrasomes offer a directional form of messaging that may profoundly alter the behaviour of immune cells within the TME. Their perspective underscores the intersection between autophagy and extracellular signalling, raising the possibility that manipulating EVs could restore immune surveillance in cancer.

The role of autophagy in modulating TME is further explored by Zhong et al. in chronic myeloid leukaemia (CML). By integrating transcriptomic and machine learning approaches, the authors classify patients based on autophagy gene expression. Their study identifies two molecular subtypes with distinct immune microenvironments, cytokine profiles, and responses to tyrosine kinase inhibitors. Interestingly, low autophagy scores correlate with immune evasion (e.g., increased PD-1 expression), while high scores associate with Treg infiltration and heightened cytokine activity. Importantly, three autophagy-related genes (*i.e.* FOXO1, TUSC1, and ATG4A) emerge as potential diagnostic markers, highlighting that autophagy is valuable as a biomarker for disease stratification.

Finally, in a different context, Yu et al. provide a detailed look into neuroinflammation following subarachnoid haemorrhage (SAH), focusing on the bidirectional communication between astrocytes and microglia. Both cell types are major players in the neuroimmune landscape and are regulated by damage-associated molecular patterns (DAMPs) and inflammatory mediators. Autophagy-related processes contribute to the activation and polarization of these cells, influencing outcomes such as blood-brain barrier integrity and neurovascular coupling. The authors advocate for targeting astrocyte-microglia crosstalk to mitigate secondary brain injury in SAH, again highlighting autophagy as a therapeutic axis to modulate inflammation also in the central nervous system.

**Figure 1:**
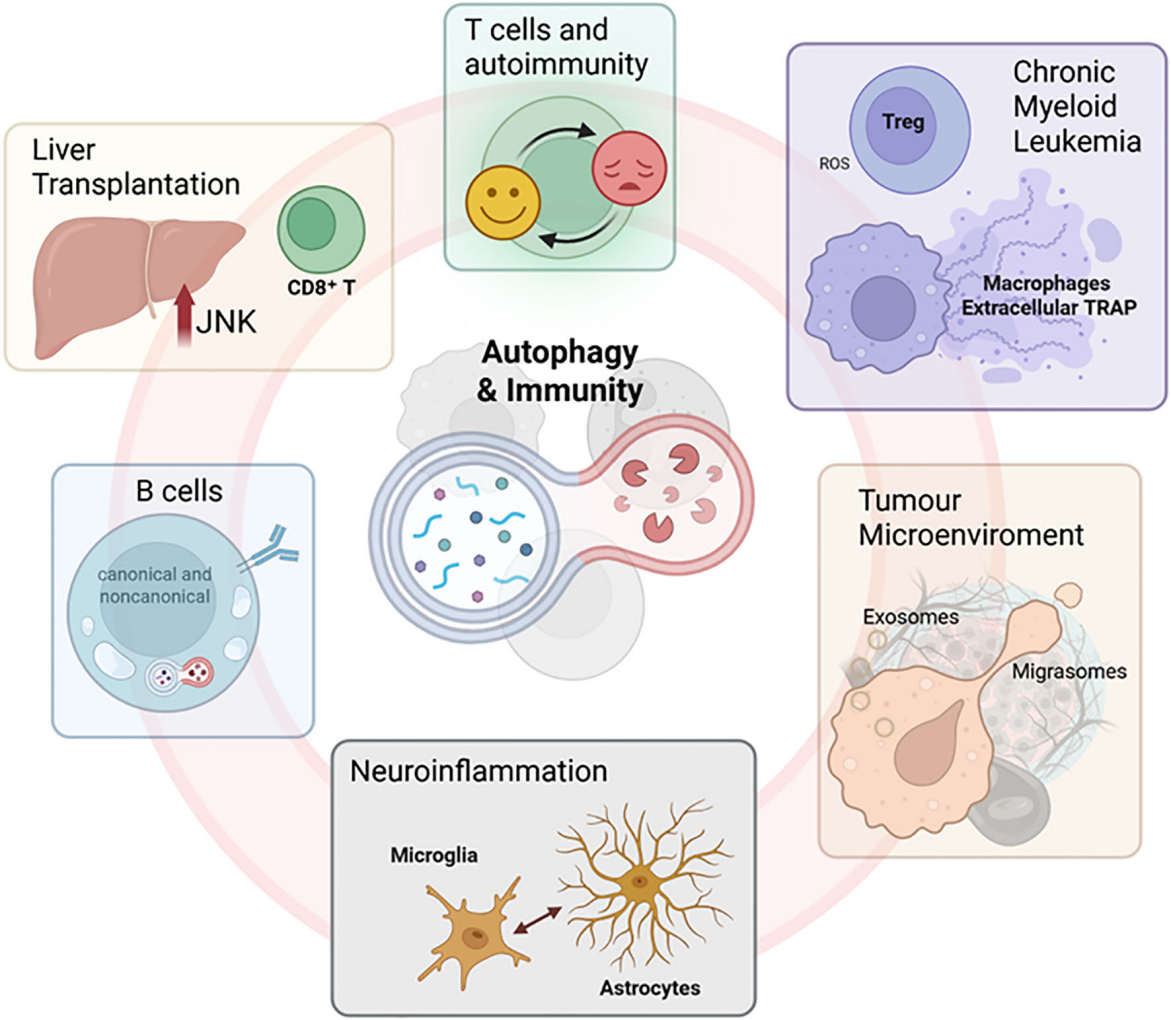
Autophagy and immune communication: From intracellular recycling to intercellular signalling in health and disease. Representative images illustrating the main concepts explored in the Research Topic. Created in BioRender. Antonioli, M. (2025) https://BioRender.com/a965tcf.

Taken together, these seven contributions illustrate that autophagy is a dynamic and versatile system capable of regulating immune function across various disease contexts (**Figure 1**). Whether enhancing T cell survival, shaping B cell memory, facilitating vesicular communication, driving neuroinflammation, or enabling extracellular trap formation, autophagy emerges as a central hub in the immune landscape. Moreover, the convergence of autophagy with other biological systems, such as signalling pathways (e.g., JNK, TLR4), vesicle trafficking (e.g., ESCRT, CMA), and metabolic adaptation, highlights its integrative role in health and disease. From a translational standpoint, these studies pave the way for autophagy-based immunotherapies that are cell-type specific and disease-tailored.

Future research will need to address several open issues, including how autophagy can be precisely modulated in specific immune cell types without off-target effects, the temporal dynamics of autophagy activation across disease stages, and whether autophagy-related biomarkers can inform patient stratification and therapeutic decisions. Importantly, understanding how the pathological environment and cell-cell communication cooperate to influence the dual role of autophagy will be crucial for identifying novel targets. The answers to these questions will shape the next generation of immune-targeted therapies.

In conclusion, autophagy has transcended its classical definition as a survival mechanism to become a critical regulator of immune cell function, intercellular communication, and disease progression. The work presented in these seven articles not only deepens our understanding of immunobiology but also reinforces the therapeutic and diagnostic promises of targeting autophagy across multiple clinical territories.

